# Optimization of variable fluorescence measurements of phytoplankton communities with cyanobacteria

**DOI:** 10.1007/s11120-012-9729-6

**Published:** 2012-03-09

**Authors:** Stefan G. H. Simis, Yannick Huot, Marcel Babin, Jukka Seppälä, Liisa Metsamaa

**Affiliations:** 1 Finnish Environment Institute SYKE, Marine Research Centre, Erik Palménin Aukio 1, 00560 Helsinki, Finland; 2 Département de géomatique appliquée, Université de Sherbrooke, 2500 boulevard de l’Unversité, Sherbrooke, QC Canada; 3 Laboratoire d’Océanographie de Villefranche, B.P. 8, Villefranche-sur-Mer, Cedex, France; 4 Takuvik Joint International Laboratory, Université Laval and CNRS, 1045 ave de la Médecine, Québec, QC G1V 0A6 Canada; 5 Estonian Marine Institute, University of Tartu, Mäealuse 14, 12618 Tallinn, Estonia

**Keywords:** Phytoplankton, Cyanobacteria, Algae, Variable fluorescence, Excitation–emission matrix, Fluorometry, Photosynthesis

## Abstract

Excitation–emission fluorescence matrices of phytoplankton communities were simulated from laboratory-grown algae and cyanobacteria cultures, to define the optical configurations of theoretical fluorometers that either minimize or maximize the representation of these phytoplankton groups in community variable fluorescence measurements. Excitation sources that match the photosystem II (PSII) action spectrum of cyanobacteria do not necessarily lead to equal representation of cyanobacteria in community fluorescence. In communities with an equal share of algae and cyanobacteria, inducible PSII fluorescence in algae can be retrieved from community fluorescence under blue excitation (450–470 nm) with high accuracy (*R* ^2^ = 1.00). The highest correlation between community and cyanobacterial variable fluorescence is obtained under orange-red excitation in the 590–650 nm range (*R* ^2^ = 0.54). Gaussian band decomposition reveals that in the presence of cyanobacteria, the emission detection slit must be narrow (up to 10 nm) and centred on PSII chlorophyll-*a* emission (~683 nm) to avoid severe dampening of the signal by weakly variable phycobilisomal fluorescence and non-variable photosystem I fluorescence. When these optimizations of the optical configuration of the fluorometer are followed, both cyanobacterial and algal cultures in nutrient replete exponential growth exhibit values of the maximum quantum yield of charge separation in PSII in the range of 0.65–0.7.

## Introduction

Differences in pigmentation are used to discriminate taxonomic phytoplankton groups in applications ranging from microscopy to remote sensing of water colour. The highest level of pigment discrimination between phytoplankton groups is found between prokaryotic cyanobacteria and the vast majority of algal taxa. Chlorophylls and carotenoids are dominant in algae, while phycobilipigments (phycoerythrin, phycoerythrocyanin, phycocyanin and allophycocyanin) are the main light harvesting pigments in cyanobacteria (prochlorophytes excepted) and red algae. Phycobilipigments extend the absorption of light to the green-orange part of the visible spectrum that is left unused by the algal groups. This spectral domain overlaps with the deepest penetration of solar irradiance in inland and coastal waters where turbidity and/or the concentration of coloured dissolved organic matter is high, yielding an advantage in light-harvesting at depth to phycobilin-containing species (Pick 1991; Stomp et al*.* 2007).

Owing to the differences in pigmentation between the major phytoplankton groups, absorption and fluorescence techniques can be used to interpret biomass at the community and sub-community level (Yentsch and Yentsch 1979; Kolbowski and Schreiber 1995; Beutler et al*.* 2002; Millie et al*.* 2002; Beutler et al*.* 2003; Seppälä and Olli 2008). In vivo chlorophyll *a* (Chl*a*) fluorescence is a widely used proxy of phytoplankton biomass, a non-intrusive measurement that can be carried out with high spatial resolution (Lorenzen 1966; Kiefer 1973) under the assumption that the Chl*a* fluorescence yield is constant. When excited with blue light, Chl*a* fluorescence per unit concentration in cyanobacteria tends, however, to be up to an order of magnitude lower than in algae, which results in erroneous biomass estimates unless corrected for (Vincent 1983; Seppälä et al*.* 2007). The distribution of Chl*a* between photosystems I and II (PSI, PSII) is fundamentally different in these phytoplankton groups (Johnsen and Sakshaug 1996, 2007), and requires consideration in all aspects of phytoplankton community fluorescence measurements.

Variable fluorescence methods relate the rise of fluorescence that occurs with ‘closure’ of PSII centres under saturating illumination to energy flow in PSII (Kautsky and Hirsch 1931; Genty et al. 1989). Closed reaction centres cannot use the energy absorbed in the photosystem antennae for photochemistry and emit at least part of the excess energy as fluorescence (e.g. Gilmore and Govindjee 1999). Saturating light conditions can be induced by generating intense light pulses, such as used in pulse-amplitude modulation (PAM), pump-and-probe and fast-repetition rate fluorescence (FRRF) techniques. These methods are designed to measure both the minimum (*F*_0_, before closure of the reaction centres and after dark-adapting the sample) and maximum inducible (*F*_m_, reaction centres closed) level of fluorescence. The variable (inducible) part of fluorescence is expressed as *F*_v_ = *F*_m_ – *F*_0_, and when normalized to *F*_m_ (*F*_v_/*F*_m_) presents a measure of the maximum quantum yield of charge separation at PSII. Under ambient light conditions, the operational quantum yield of PSII (*F*_v_′/*F*_m_′) is obtained instead. Both parameters are useful as they respond to nutrient limitation, excess light or transiently when growth conditions change. A combination of dark- and light adapted measurements can be used to determine the electron transport rate under known irradiance(s), which can in turn be used to model primary production (Kolber and Falkowski 1993). The current work focuses on the experimental manipulation and spectral measurement of dark-adapted *F*_v_/*F*_m_. The use of this parameter in higher level applications is discussed at length in recent reviews of literature on the subject (Suggett et al. 2004, Huot and Babin 2010).

Advances in light-emitting diode (LED) manufacturing have led to the availability of narrow-band, high-power excitation light sources of high efficiency and stability. Their rapid flash capability and high output makes them the light source of choice for FRRF protocols and for PAM applications that require a small footprint. In FRRF, microsecond flashlets provide a saturating flash train within a single turnover period of PSII (<100–150 μs). PAM-type fluorometers have been developed with a combination of light sources of different colours for some time. FRRF instruments were until very recently limited to the use of LEDs of one colour in order to produce sufficiently bright flashlets. Blue light sources have been chosen to provide overlap with the absorption by Chl*a* and accessory photosynthetic pigments in algae, but do not overlap with cyanobacterial phycobilipigment absorption (Johnsen and Sakshaug 2007). Recent studies have shown that blue-light equipped FRRF instruments are relatively insensitive to the presence of cyanobacteria, if these do not possess short-wavelength forms of phycoerythrin (Raateoja et al. 2004; Suggett et al. 2004). While *F*_v_/*F*_m_ can be recorded from cyanobacteria using blue excitation as long as the light source can saturate PSII, the intensity of the fluorescence is relatively low compared to algae. Variable fluorescence of cyanobacteria can alternatively be assessed from orange or red excitation sources that excite the phycobilipigments in cyanobacteria (Schubert et al. 1989). Now that LEDs are available at the brightness required by FRRF instruments, this concept stands to be adapted to the FRRF range of instrumentation. Studies on the optimization of the variable fluorescence measurement towards unbiased representation of the phytoplankton community, are therefore overdue.

In this study, we address the representation of cyanobacteria and algae in community (variable) fluorescence measurements with special emphasis on narrow-band excitation sources. Our focus is on cyanobacteria with a pigment profile that results in low fluorescence under blue light. Most coastal and freshwater cyanobacteria belong to this group, whereas common clear-water species that produce phycourobilin-rich forms of phycoerythrin have stronger fluorescence with blue excitation. We analyse fluorescence excitation–emission matrices of cultures that are subjected to various treatments of light and nutrient availability. These fluorescence matrices are used to simulate variable fluorescence of mixed algal and cyanobacterial communities from which statistical analyses of the relation between community and subcommunity variable fluorescence follows. We describe the optimal optical configuration (excitation–emission waveband pairs) to obtain *F*_v_/*F*_m_ values that represent a community cross section regardless of the share of cyanobacteria in the community. The excitation–emission waveband pairs that result in the best correspondence of community *F*_v_/*F*_m_ measurements with either the cyanobacterial or the algal subpopulation are also determined.

In previous studies, healthy cyanobacteria have reported maximum *F*_v_/*F*_m_ in the order of 0.3–0.5 and seldom >0.6 (Raateoja et al. 2004; Suggett et al. 2009). This is markedly lower than reported for algae (0.65) and higher plants (near 0.8). Low *F*_v_/*F*_m_ in healthy cells can be a measurement artefact when the light source does not provide sufficient intensity to saturate PSII (Raateoja et al. 2004). The solution is then to be found in the use of excitation wavebands that better match the photosynthetic action spectrum of the sample. It has also been suggested that phycobilipigment fluorescence can elevate *F*_0_ in the PSII Chl*a* fluorescence band, and thus reduce observed *F*_v_/*F*_m_ (Campbell et al. 1996, 1998). Interestingly, this latter effect prevails under excitation with blue light, which incites only weak fluorescence from phycobilisome (PBS) pigments. To resolve this issue, we use Gaussian band decomposition of fluorescence emission spectra to determine the extent to which PSII *F*_0_ and *F*_m_ are offset by phycobilipigment fluorescence. We then show how the excitation and emission slits of the fluorometer can be optimized to exclude fluorescence from phycobilisomal and PSI pigments, yielding cyanobacterial *F*_v_/*F*_m_ values in the same range as observed in algae.

## Methods

### Phytoplankton cultures

The algal species included in this study were the chlorophyte *Brachiomonas submarina* TV15 and the diatom *Thalassiosira pseudonana* TV5 from the Tvärminne culture collection (TV, University of Helsinki, Hällfors and Hällfors 1992). Cyanobacterial strains included the closely related phycocyanin-rich and phycoerythrin-rich picocyanobacteria strains *Synechococcus* sp. CCY9201 and CCY9202 (Culture Collection Department of Marine Microbiology, NIOO-KNAW, The Netherlands), both isolated from the Baltic Sea (Ernst et al. 2003). Further, two morphologically and optically highly similar strains of the filamentous bloom-forming *Nodularia spumigena* were included: strain HEM from University of Helsinki, Microbiology division (Sivonen et al*.* 1989), and one with an undocumented culturing history that we conservatively annotate *Nodularia* sp. from the TV collection. All species are common in the Baltic Sea.

Nutrient replete cultures were grown on sterile modified BG-11 media with salinity adjusted to the Baltic Sea at 8.3 g NaCl L^−1^, pH = 7.4, and added vitamin B12 (0.02 μg L^−1^). Silicate was added to the diatom cultures at 0.044 g Na_2_SiO_3_·5H_2_O L^−1^. BG11 medium is rich in nitrate (N:P approximately 100:1), so cultures that were left to grow and age in a particular batch were expected to eventually become starved of phosphorous. To induce nitrogen starvation instead, selected cultures were periodically refreshed with medium with reduced (10%) nitrate (N treatment) or no nitrate (-N treatment). These treatments were expected to induce fixation of elemental nitrogen in the *Nodularia* strains. Light conditions were 12/12 h light/dark from fluorescent tubes at low/medium/high light treatments of 20, 70 or 350 μmol photons m^−2^ s^−1^, respectively, using green filters to mimic the Baltic Sea environment in the low and medium light levels. The green filters also increased production of phycobilipigments, particularly in the *Synechococcus* strains. The cultures were kept in suspension by daily gentle mixing, and bubbling with filtered air for 15 min every hour. The complete combination of treatments and sampling times (i.e. aging of the cultures) is presented in Table 1. Cultures that exhibited no growth after up to 2 weeks were removed from the experiment. Cultures that underwent significant visual changes were sampled more than once. The different treatments resulted in a total of 31 sampling events of cyanobacterial cultures and 15 sampling events of the algal cultures.

**Table 1:** Culturing conditions

Cultured species	Culturing conditions (light, nutrients)				
	20, +N	70, +N	70, N	350, N	350, −N
*Synechococcus* sp. CCY9201^a^	5, 8	7, 8		8	2
*Synechococcus* sp. CCY9202^a^	12, 14, 19	5, 8, 11,12		8	
*Nodularia spumigena* HEM^b^	14, 17	7, 14, 17	12, 21	11, 14, 16	
*Nodularia* sp.^c^		7, 13, 17	12, 21	11, 23	
*Brachiomonas submarina* TV15^c^		7, 17, 11, 34		8	2, 7
*Thalassiosira pseudonana* TV5^c^		12, 13, 14, 17, 24, 34		7	2

The numbers under each growth regime indicate the time (days) that the respective culture was left to grow/age after inoculation, before sampling took place. Growth light intensities (values in column headers) have units μmol photons m^−2^ s^−1^. Nitrogen additions are indicated with +N, N, −N for nitrogen replete, nitrogen limited and nitrogen deplete conditions

^a^Ernst et al. (2003)

^b^Sivonen et al. (1989)

^c^Hällfors and Hällfors (1992)

### Absorption measurements

Spectrophotometric measurements were carried out using a PerkinElmer (Waltham, MA, USA) model Lambda650 spectrophotometer equipped with a 150-mm integrating sphere. The samples were placed in a 10-mm quartz cuvette at the front entrance of the sphere. Cultures were diluted as necessary to measure in the range where optical density (OD) was linear with dilution. In this configuration, the measured OD can be assumed proportional to absorption and backscattering. A baseline equal to OD at 800 nm was subtracted to correct for backscatter. Purified, filtered water was used as a blank reference. Absorption (*a*) was derived from the OD measurements using *a*(λ) = 2.303 × OD_bc_(λ)/0.01, where the factor 2.303 serves to convert from a 10-based to a natural logarithm, OD_bc_(λ) is the baseline-corrected OD at wavelength λ, and 0.01 is the path length of the cuvette in meters.

### Fluorescence measurements

All spectral fluorescence measurements were carried out after placing samples in low light (<10 μmol photons m^−2^ s^−1^) for at least 0.5 h. Excitation/emission matrices of fluorescence were recorded for the diluted (see below) samples in a 10-mm quartz cuvette in a Varian Cary Eclipse (Agilent, Santa Clara, CA, USA) fluorometer. Emission was scanned from 600 to 750 nm at 1-nm intervals and 10-nm band width, while excitation was produced with a Xenon flash lamp in 10-nm bands, at 10-nm intervals from 400 to 650 nm. It is essential for the proper determination of *F*_v_/*F*_m_ that our *F*_0_ measurements were not disturbed by fluorescence induction in any part of the excitation–emission matrix, particularly in the case of cyanobacteria which are known to undergo state transitions at very low light intensity. The excitation beam was attenuated to 25% using neutral density filter as a precaution. A selection of cultures tested before the start of the experiment showed that increasing the attenuation of the excitation light did not change the observed *F*_v_/*F*_m_ or the spectral shape of *F*_0_ emission. Repeated excitation–emission matrix measurements also gave identical results. This empirical evidence, although circumstantial, suggests that neither the intensity nor the period of illumination prevented the measurement of *F*_0_ or *F*_v_/*F*_m_. These assumptions are also supported in a theoretical sense, when we consider properties of the excitation light source and sample placement: the Xenon flash lamp produces 2–5 μs half-width pulses at 80 Hz. This flash interval (>12 ms) allows relaxation of PSII between flashes. With a microspherical PAR sensor in the focused excitation beam centred in a 10-nm wide band at 420 nm (the peak wavelength of the lamp), we derived a photon density in the order of 0.01 μmol photons m^−2^ flash^−1^ which should not excite above *F*_0_ (see Biggins and Bruce 1989; Babin et al*.* 1995). Finally, the excitation beam illuminated approximately 6% of the cell suspension at any given time, while the sample was continuously stirred. These considerations support our assumption that no significant build-up of fluorescence above *F*_0_ occurred, and that multiple turnover did not induce transitions to state I. Indeed, the placement of neutral density filter was in all likelihood not required. *F*_m_ was measured by treating the samples with 3-(3,4-dichlorophenyl)-1,1-dimethylurea (DCMU, Sigma-Aldrich) to a final concentration of 30 μM. By blocking the electron acceptor side of PSII, DCMU causes a fluorescence rise to *F*_m_. Excitation–emission matrices were quantum-corrected following Kopf and Heinze (1984), accounting for spectral dependency of the light source and detector, and corrected for the spectral attenuation of the neutral density filter. The spectral resolution and detector sensitivity allowed scanning of one excitation–emission matrix in approximately 10 min. Blank spectra (culture medium) were measured daily and subtracted from *F*_0_ and *F*_m_ spectra.

### Dilutions and normalization

The fluorescence data used in our analyses was normalized to absorption to correct for differences in cell density and pigmentation between cultures. The normalization was achieved by diluting the stock cultures instead of scaling measured fluorescence intensities. While this approach may cause some dilution errors, it also minimizes the effects of multiple scattering and reabsorption of fluoresced light that may be present in dense cultures. Variability in the Chl*a*-specific absorption at 675 nm is fairly limited in algal cultures compared to cyanobacteria, because the latter exhibit more prominent overlap between phycobilipigment and Chl*a* absorption in the red spectral domain. In contrast, variability around the blue Chl*a* absorption peak is relatively limited in cyanobacteria cultures and introduced foremost by photoprotective carotenoids. To prevent these differences in pigmentation from creating biases in our fluorescence data set, we used different absorption measures for the dilution of either group. The dilution target for algal cultures was set at *a*(675) = 5.0 m^−1^. Cyanobacterial samples were diluted to match *a*(437) = 9.9 m^−1^, which resulted in an average *a*(675) of 4.6 and standard deviation of 1.1 m^−1^ over all cyanobacteria cultures. The fluorescence signals obtained from the cultures diluted in this way were not scaled further and are henceforth referred to as fluorescence normalized to *a*(675) or absorption-normalized fluorescence. In a few cases where the stock culture had a lower OD than the target value, corresponding fluorescence values were proportionally adjusted. All dilutions were made using BG-11 growth medium lacking nitrate and phosphate to avoid replenishment of nutrient-starved cultures. Community fluorescence excitation–emission matrices (*F*_0_, *F*_m_, and derived *F*_v_/*F*_m_) were constructed by addition of the absorption-normalized fluorescence signals.

## Results

### Spectral characteristics of absorption and fluorescence

**Fig. 1:**
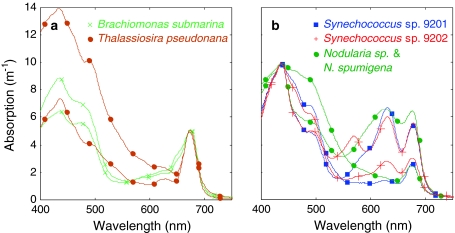
Diversity of absorption spectra of the cultures used to simulate community fluorescence. Only the absorption spectra of the **a** algal and **b** cyanobacterial cultures representing highest and lowest blue-to-red absorption ratios are shown for each of the cultures species

**Fig. 2:**
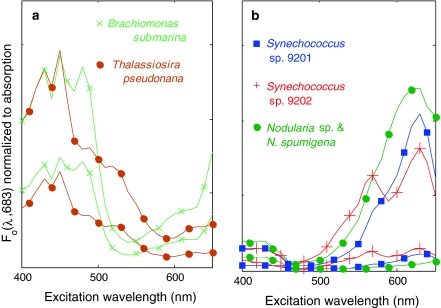
Diversity in fluorescence excitation spectra (*F*_0_, emission 683 nm, spectra normalized to absorption as described under ‘Methods’) of the **a** algal and **b** cyanobacterial cultures used to simulate community fluorescence. Only the brightest and weakest fluorescing examples of each species are shown

Gradual nutrient starvation, variable light exposure and sampling at various moments during culturing led to considerable variability in absorption and fluorescence. This variability is illustrated in Fig. 1 for spectral absorption and in Fig. 2 for fluorescence excitation with Chl*a* emission measured at 683 nm. For each species that was included in our analysis Fig. 1 shows the absorption spectra of the extreme cases, in terms of the blue-to-red absorption ratio. These absorption spectra correspond to the same diluted samples that were used to measure fluorescence (Fig. 2). Samples of *Synechococcus* sp. CCY9202 show the characteristic absorption peak of phycoerythrin (around 560 nm) as their dominant accessory pigment. The other cyanobacteria cultures showed dominant accessory photosynthetic pigment absorption at longer wavelengths, in *Nodularia* matching the absorption characteristics of phycocyanin possibly mixed with phycoerythrocyanin (600–630 nm). Phycocyanin (~615 nm) showed as the dominant pigment in *Synechococcus* sp. CCY9201. The absorption by accessory photosynthetic pigments chlorophyll *b* (~650 nm) and chlorophyll *c* (~630 nm) can be recognized in the red part of absorption spectra of respectively the chlorophyte *Brachiomonas submarina* and the diatom *Thalassiosira pseudonana*.

The range of variation in spectral absorption in algae and cyanobacteria cultures was comparable in terms of the extremes shown in Fig. 1a, b, respectively. Nevertheless, the cyanobacteria cultures were more evenly spread between these extremes than the algae cultures. High light (350 μmol m^−2^ s^−1^) treatment resulted in increased blue-to-red absorption ratios in the algae cultures, possibly due to the enhanced production of photoprotective pigment absorbing blue light. All cyanobacteria responded to low (20 μmol m^−2^ s^−1^) light treatment with increased pigment production and pronounced absorption features of the phycobilipigments. Chlorosis occurred in the cyanobacteria cultures under high light treatment and increasingly with nitrogen starvation. *Nodularia* sp. is known to fix elemental nitrogen and its accessory pigment production appeared to recover after an initial period of reduced absorption and slow growth under nitrogen starvation. *Synechococcus* sp. CCY9202, adapted to low light environments (Wood 1985; Pick 1991), only showed increasing absorption under low light, while all other cyanobacteria showed prominent accessory pigment features under both low and medium light intensity (70 μmol m^−2^ s^−1^).

The fluorescence excitation spectra for Chl*a* fluorescence given in Fig. 2 (as *F*_0_(λ_ex_,683)) can be interpreted as photosynthetic absorption spectra of PSII. A large difference in the blue versus red light harvesting for PSII is apparent between algae and cyanobacteria when comparing absorption in Fig. 1 to the PSII fluorescence in Fig. 2. The prominent role of Chl*a* in light harvesting for PSII in algae, visible in the blue around 440 nm, is nearly absent in the cyanobacterial strains, where only a small share of Chl*a* is connected to PSII (Johnsen and Sakshaug 1996, 2007). The algal species further reveal light harvesting for PSII in the area of maximum absorption by accessory pigments in the 460–480 nm range: fluorescence resulting from excitation at 470–480 and at 650 nm in *B. submarina* may be attributed to Chlorophyll *b*, whereas in *T. pseudonana*, excitation at 460–470 and 630 nm would be due to Chlorophyll *c* and excitation at 530–540 nm due to fucoxanthin. Between the two algal species, affinity for red light was higher in *B. submarina,* in some cases exceeding fluorescence from red excitation found in cyanobacterial cultures that were nutrient starved. The Chl*a* fluorescence excitation features found in the cyanobacterial cultures matched the absorption peaks of phycobilipigments given above. Between the cyanobacterial cultures *Nodularia* showed the highest absorption-normalized fluorescence under blue illumination. Cyanobacteria with urobilin-rich phycoerythrin, which may absorb short-wavelength light down to 490 nm and are common in clear water environments, were not included in our data set.

**Fig. 3:**
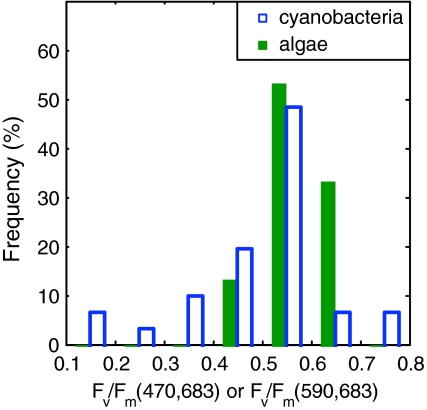
Histograms of *F*_v_/*F*_m_ for the cultures used in this study. *F*_v_/*F*_m_(470 and 683) are shown for the algal cultures and *F*_v_/*F*_m_(590 and 683) are shown for the cyanobacteria, corresponding to sections of the excitation–emission matrices with the highest fluorescence signal in either group

The variability in *F*_v_/*F*_m_ of the species used in this study is shown as histograms in Fig. 3. The excitation bands to describe *F*_v_/*F*_m_ in algae and cyanobacteria were selected to match peak areas in the excitation spectra (Fig. 2). *F*_v_/*F*_m_ of the algae is shown for excitation at 470 nm, cyanobacterial *F*_v_/*F*_m_ at 590 nm (both for 10-nm bandwidth). The emission was measured at 683 nm (10-nm bandwidth) for both groups. Maximum *F*_v_/*F*_m_ in the order of 0.65 are common in phytoplankton studies (but see Samson et al. 1999; Suggett et al. 2004; Vredenberg et al. 2009). The majority of cultures included in our analyses showed *F*_v_/*F*_m_ in the 0.45–0.65 range, while the range of *F*_v_/*F*_m_ in cyanobacterial cultures was wider (0.1–0.7) than that of algal cultures (0.4–0.7). The top range of these *F*_v_/*F*_m_ values measured in cyanobacteria exceed those commonly found in literature, where values for healthy cultures are usually in the 0.3–0.5 range (but see Raateoja et al. 2004; Suggett et al. 2009). Lower *F*_v_/*F*_m_ in cyanobacteria has been attributed to incomplete saturation of PSII in FRRF studies (Raateoja et al. 2004), and to dampening of the variable fluorescence by an offset of *F*_0_ caused by fluorescing phycobilipigments (Campbell et al. 1996, 1998), which is discussed further below.

**Fig. 4:**
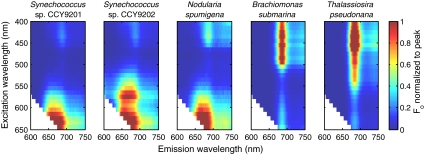
*F*_0_ excitation–emission matrices of a culture of each of the species included in this study. These cultures were sampled under nutrient replete growth conditions and had *F*_v_/*F*_m_ values of 0.6–0.7. The matrices are normalized to the spectral maximum to facilitate comparison of spectral differences between the different species

**Fig. 5:**
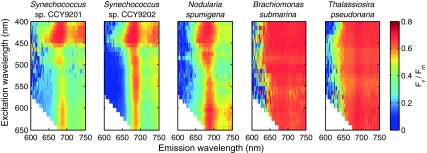
*F*_v_/*F*_m_ excitation–emission matrices for the cultures shown in Fig. 4

Spectral fluorescence excitation and emission properties of the phytoplankton strains used in this study are further illustrated by excitation–emission *F*_0_ and *F*_v_/*F*_m_ matrices in Figs. 4 and 5, respectively. The matrices shown here are representative for optimal growth conditions (low to medium light intensity depending on species, nutrient replete growth media and sampled during the exponential growth phase). The *F*_0_ fluorescence matrices show prominent fluorescence emission features in cyanobacteria under orange-red excitation that are characteristic of PBS (fluorescence emission around 650 nm from allophycocyanin) and Chl*a* (680 nm) pigments. In contrast, the algal strains reflect the absorption of light by chlorophylls and carotenoids in the blue-green spectral region with a sharply defined emission related to Chl*a* fluorescence.

Despite the sharp distinction in *F*_0_ profiles observed between algae and cyanobacteria, *F*_v_/*F*_m_ matrices (Fig. 5) show relatively constant *F*_v_/*F*_m_ in the Chl*a* emission band in both cyanobacteria and algae. For algal fluorescence, the variable component extends along the whole excitation spectrum for emission from ~650 to at least 750 nm (the maximum measured). The excitation–emission patterns for the cyanobacterial cultures show a smoother transition from low to high *F*_v_/*F*_m_ when emission wavelength increases towards the maximum of PSII Chl*a* (680–690 nm), but a sharp drop of *F*_v_/*F*_m_ at longer emission wavelengths (>700 nm). These features can respectively be explained by a variable component to PBS fluorescence (discussed further below), and the allocation of most Chl*a* molecules to the non-variable PSI in cyanobacteria (Johnsen and Sakshaug 1996, 2007). The feature-rich *F*_v_/*F*_m_ profile of cyanobacteria implies that the spectral location and bandwidth of emission detection can have a major influence on readings of *F*_v_/*F*_m_, when we target Chl*a* emission in cyanobacteria. Optimization of detector slit spectral position and bandwidth for equivalent readings of *F*_v_/*F*_m_ in cyanobacteria and algae are discussed in more detail below.

### Simulations of community fluorescence

*F*_v_/*F*_m_ is used to assess the maximum efficiency of PSII in dark-acclimated cells. *F*_v_/*F*_m_ can be expressed for all waveband pairs in the excitation/emission matrix, and because the fluorescence excitation–emission matrices of algae and cyanobacteria differ prominently (Fig. 5) it is useful to inspect excitation–emission matrices of phytoplankton communities in search of features that can be uniquely attributed to specific phytoplankton groups. This analysis requires knowledge of the spectral fluorescence properties as well as the inducible fluorescence of all species represented in a community. These requirements cannot be met when analysing natural samples consisting of multiple species contributing unique signals to bulk fluorescence. Instead, we simulated community fluorescence from the excitation–emission *F*_0_ and *F*_m_ measurements of individual cultures. We constructed community fluorescence excitation–emission matrices, each consisting of a single algal and a single cyanobacterial species. Different culturing conditions and different times of sampling (Table 1) resulted in 15 algal and 31 cyanobacterial input matrices and 465 unique combinations. With this large number of combined excitation–emission matrices for which *F*_0_ and *F*_m_ (and thus *F*_v_/*F*_m_) were available, it was possible to perform statistical analyses of the relation between community and algal or cyanobacterial *F*_v_/*F*_m_. This evaluation was carried out for individual excitation–emission waveband pairs.

Although *F*_v_/*F*_m_ can be measured for any waveband pair in an excitation–emission matrix, we can only interpret the variable fluorescence that originates from Chl*a* in PSII (at 680–690 nm) in terms of the electron flux that fuels photosynthesis. We therefore examine the simulated community *F*_v_/*F*_m_ excitation–emission matrices against the PSII Chl*a* *F*_v_/*F*_m_ values of their algal and cyanobacterial fractions. To identify the contribution from the algal or cyanobacterial fraction *F*_v_/*F*_m_ to community *F*_v_/*F*_m_, the reference excitation–emission pair (both denoted λ_ref_) for cyanobacteria and algae are chosen from regions of the excitation spectrum of Chl*a* fluorescence where we encounter a high fluorescence yield and strong variable fluorescence. We selected λ_ref_ = 470 and 590 nm of 10-nm width for algae and cyanobacteria, respectively. Choosing different λ_ref_ values within the blue and orange-red excitation domain does not lead to significantly different results. The 470-nm band is located between the absorption maxima of Chl*a* and accessory chlorophylls in the algal cultures, the latter are not present in cyanobacteria. The 590-nm band (10-nm wide) is chosen to excite cyanobacterial phycobilipigments that absorb in the 550–630 nm domain. The emission waveband for the reference *F*_v_/*F*_m_ is centred at 683 nm and has a width of 10 nm.

**Fig. 6:**
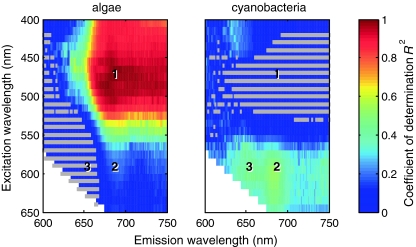
*R* ^2^-values (colour scale) for linear least-squares regression of *F*_v_/*F*_m_(λ_ex_,λ_em_) in simulated communities against *F*_v_/*F*_m_ of their **a** algal and **b** cyanobacterial subpopulations. Each *R* ^2^ value represents the regression of all 465 communities. The regression of community *F*_v_/*F*_m_(λ_ex_,λ_em_) was carried out against *F*_v_/*F*_m_(470,683) of algal subpopulations and against *F*_v_/*F*_m_(590,683) of cyanobacterial subpopulations. *Grey markers* indicate a poor fit (*p* > 0.001) of the regression model to the data. *Numeric markers* refer to excitation/emission pairs for which case plots are given in Fig. 8a–c

Owing to the large number of simulated communities, we are able to highlight the influence of algal and cyanobacterial signals in community *F*_v_/*F*_m_(λ_ex_,λ_em_) using regression statistics. The matrices of the coefficient of determination (*R* ^2^) of community *F*_v_/*F*_m_(λ_ex_,λ_em_) against *F*_v_/*F*_m_(λ_ref_,683) of their algal and cyanobacterial subpopulations are given in Fig. 6. Three excitation/emission regions (marked 1–3 in Fig. 6 and subsequent figures) are identified as showing a correlation between community, and algal or cyanobacterial *F*_v_/*F*_m_ that is higher than in adjacent areas:

- Region 1 shows *R* ^2^ close to 1 between community and algal *F*_v_/*F*_m_ (and consequently a *R* ^2^ near 0 with the cyanobacterial fraction), under blue excitation in a wide emission band that includes Chl*a* fluorescence and extends into the range of mixed PSI/PSII fluorescence at near-infrared wavelengths.
- Region 2 is for excitation near 600 nm and emission in the Chl*a* fluorescence band near 683 nm and returns *R* ^2^ above 0.5 for cyanobacteria but 0.2 for algae. In contrast to the correlation with algae in region 1, the excitation range with a high correlation for cyanobacterial *F*_v_/*F*_m_ does not extend into the near-infrared.
- Region 3, similarly to region 2, is found under orange/red excitation, but in the emission range of phycobilipigments (620–650 nm). In this spectral domain, *R* ^2^ is greater than 0.4 for cyanobacteria and near 0 for algae, as no algal pigments fluoresce around 650 nm (Fig. 4). While the presence of highly fluorescent phycobilipigments in cyanobacteria explains strong fluorescence between 600 and 650 nm, the correlation (*R* ^2^ > 0.4) to variable fluorescence from PSII Chl*a* is not straightforward, as it has commonly been assumed that phycobilipigment fluorescence is not variable (but see discussion below, and Küpper et al. 2009; Kana et al. 2009). We note that the presence of algae in the community does not influence this result as regression of *F*_v_/*F*_m_(590,650) against *F*_v_/*F*_m_(590,683) yields the same correlation when measured from the 31 individual cyanobacterial cultures.

**Fig. 7:**
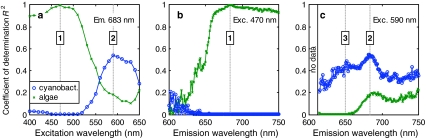
*R* ^2^ for regressions of *F*_v_/*F*_m_(λ_ex_,λ_em_) of simulated communities against *F*_v_/*F*_m_(470,683) and *F*_v_/*F*_m_(590,683) of respectively algal and cyanobacterial subpopulations. These plots represent cross sections of the excitation–emission regression matrix of Fig. 6: **a** the 683-nm emission line, **b** the 470-nm excitation line, and **c** the 590-nm excitation line. Key excitation–emission pairs are indicated by the *numeric markers* corresponding to Figs. 6 and 8

To find optimal excitation and emission pairs for the separation of cyanobacterial and algal *F*_v_/*F*_m_ in communities, we inspect the data more closely along the emission and excitation lines linked to the previously identified regions 1–3. The PSII Chl*a* emission line (683 nm, Fig. 7a) reveals that the strongest correlations of *F*_v_/*F*_m_(λ_ex_,683) with algal and cyanobacterial *F*_v_/*F*_m_ occurred upon excitation between 440–500 and 590–630 nm, respectively. The 470-nm excitation line (Fig. 7b) reveals that *F*_v_/*F*_m_(470,λ_em_), particularly for emission >650 nm, was exclusively and strongly correlated with the algal fraction of the community. The emission spectrum along the 590-nm excitation line (Fig. 7c) confirms that emission around 650 and 683 nm was best correlated with cyanobacterial *F*_v_/*F*_m_ (with *R* ^2^ in the range 0.4–0.5) and weakly correlated with algal *F*_v_/*F*_m_ in the Chl*a* emission band (*R* ^2^ < 0.2).

**Fig. 8:**
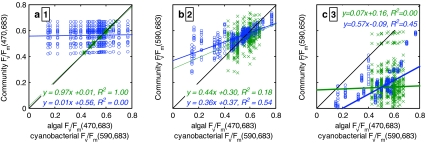
Case plots underlying the linear regression analyses of community *F*_v_/*F*_m_(λ_ex_,λ_em_) versus algal and cyanobacterial *F*_v_/*F*_m_(470,683) and *F*_v_/*F*_m_(590,683), respectively. **a**–**c** correspond to the key excitation–emission pairs highlighted with *numerical markers* in Fig. 6. **a** *F*_v_/*F*_m_(470,683), sensitive to algal but not cyanobacterial *F*_v_/*F*_m_, **b** *F*_v_/*F*_m_(590,683), with stronger correspondence to cyanobacterial compared to algal *F*_v_/*F*_m_ and **c** *F*_v_/*F*_m_(590,650), strongly related to cyanobacterial *F*_v_/*F*_m_(590,683) >0.4. *Colours and symbols* correspond to Fig. 7, *drawn black lines* mark unity. The discrete distribution of the subcommunity *F*_v_/*F*_m_ values is caused by the limited number of cultures used to simulate community *F*_v_/*F*_m_ matrices

The data underlying the optimal excitation/emission pairs identified from Figs. 6 and 7 are presented in Fig. 8 with corresponding regression statistics. Figure 8a confirms that community *F*_v_/*F*_m_(470,683) is strongly driven by the algal *F*_v_/*F*_m_ and was highly insensitive to the fluorescence of the cyanobacteria in the simulated communities. Only the case for equal absorption in the algal and cyanobacterial subpopulations is shown here, but when the community composition was skewed to 90% in favour of the cyanobacteria, community *F*_v_/*F*_m_(470,683) remained a good (relative error <10%) predictor of algal *F*_v_/*F*_m_(470,683) in 92% of cases. The fluorescence emission of the cyanobacterial fraction was too low at this excitation/emission pair to influence community variable fluorescence, even when mixed with algal cultures of low (variable) fluorescence.

Under red–orange illumination centred at 590 nm (Fig. 8b) we note a better correlation of community and cyanobacterial *F*_v_/*F*_m_ (*R* ^2^ = 0.54). The relatively low slope and high offset of this regression were clearly caused by the inclusion of cases where cyanobacterial subpopulations with low *F*_v_/*F*_m_ were mixed with algae with higher *F*_v_/*F*_m_, a result of a wider spread of *F*_v_/*F*_m_ in the cyanobacterial cultures compared to the algae (Fig. 3). The regression results for the algal fraction under emission at 590 nm were clearly worse with *R* ^2^ = 0.18.

The variable fluorescence originating from PBS pigments (*F*_v_/*F*_m_(590,650)) was lower than *F*_v_/*F*_m_(590,683) while the relation between community and cyanobacterial *F*_v_/*F*_m_ was strong for cyanobacteria cultures with *F*_v_/*F*_m_ >0.42 (Fig. 8c). Cultures with lower *F*_v_/*F*_m_ included several where phycobilipigments were bleached (primarily in high light) or broken down (primarily under nitrogen stress). If these cultures are not considered, the *R* ^2^ value for cyanobacteria improved from 0.45 to 0.76. These results suggest a tight coupling between the *F*_v_/*F*_m_ from PBS pigments and PSII Chl*a*, which is further explored in the next section. The high amount of scatter in the results comparing community *F*_v_/*F*_m_(590,650) against the algae fraction provides further indication that the variable fluorescence of cyanobacteria cultures can be observed from community *F*_v_/*F*_m_ without interference from the presence of algae.

### The nature of cyanobacterial fluorescence in the Chl*a* emission band

The emission spectra of algal cultures at room temperature have a predictable shape because their main source of fluorescence is Chl*a* located in PSII and to a much smaller extent in PSI. In cyanobacteria, we observe fluorescence in the red spectral domain from (1) PSII Chl*a* (variable), (2) PBS fluorescence (weakly variable) and (3) PSI (non-variable), where the contribution of the latter is relatively strong in cyanobacteria compared to algae. The role of PSI fluorescence in the red spectral domain is likely to be important in fluorometers that record fluorescence >700 nm (discussed below). The role of accessory PSII pigment composition on fluorescence in the PSII Chl*a* emission band and towards shorter wavelengths has received very little attention altogether and is explored here.

It has been suggested that phycobilipigments have a significant effect on the *F*_0_ signal that is otherwise attributed to Chl*a* (e.g. Campbell et al. 1996, 1998). A non-variable fluorescence source elevates *F*_0_ and *F*_m_ equally, which leads to dampening of *F*_v_/*F*_m_. We observed in the previous exercise that the PBS fluorescence does have a (weakly) variable component, which in turn should alleviate this dampening. To quantify the influence of PBS fluorescence on the variable fluorescence from PSII it is necessary to isolate *F*_0_ and *F*_m_ of the individual pigments. We decomposed *F*_0_ and *F*_m_ emission spectra of our cyanobacteria cultures into Gaussian band contributions of phycobilipigments and Chl*a*. The Gaussian decomposition allows us to express *F*_v_/*F*_m_ of each pigment component.

**Fig. 9:**
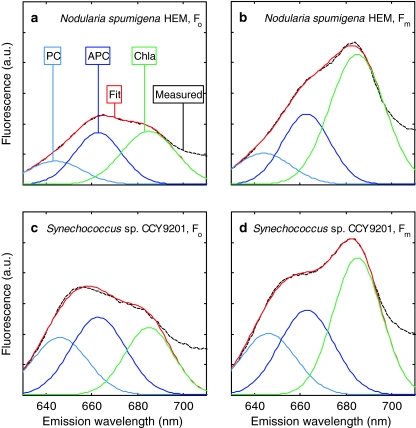
Fluorescence emission spectra at *F*_0_ and *F*_m_ of two cyanobacteria illustrating Gaussian band decomposition into the contributions of Chl*a* and phycobilipigments (see text), and the occurrence of a variable component to the fluorescence attributed to phycobilipigments. **a** *F*_0_(590,λ) of *Nodularia spumigena* HEM, **b** *F*_m_(590,λ) of *N. spumigena* HEM, **c** *F*_0_(590,λ) of *Synechococcus* sp. CCY9201, **d** *F*_m_(590,λ) of *Synechococcus* sp. CCY9201

Emission spectra were taken from the excitation–emission matrices of all cultures used in the simulations described in the previous section. We restrict ourselves to fluorescence emission between 625 and 690 nm, assuming that components of PSI and PSII that fluoresce at longer wavelengths (PSII Chl*a* at 730–740 nm, PSI Chl*a* >700 nm, c.f. Ley 1980) have minimal influence in the area around 680 nm. The emission band corresponding to excitation at 590 nm (10-nm bandwidth) was selected as it yields high fluorescence in all cyanobacteria cultures. The choice or width of the excitation band does not influence the shape of the emission spectrum, as long as the excitation band overlaps with the absorption domain of the PBS pigments that fuel PSII. An overview of the boundary conditions for fitting the model is given in Table 2. Each Gaussian curve was defined as

1 $$ F({{\uplambda}}) = \alpha \cdot {\text{e}}^{{\frac{{ - (\lambda - \beta )^{2} }}{{2\gamma^{2} }}}} $$

where *F* denotes the fluorescence at waveband λ, and *α* the magnitude, *β* the centre wavelength, and *γ* the standard deviation of the curve. We assumed no change in the value of *β* and *γ* between *F*_0_ and *F*_m_ for any given sample. The least squares difference between measured *F*_0_ or *F*_m_ (625–690 nm) and the fluorescence of three pigment components (phycocyanin, allophycocyanin and Chl*a*) was minimized, allowing up to 2.5% deviation of the fit at the pigment fluorescence maxima. Fitted spectra of *N.* *spumigena* HEM and *Synechococcus* sp. 9201 are presented in Fig. 9 as examples of the fit results. The fit results for *N.* *spumigena* HEM (Fig. 9a, b) clearly show the variable component of fluorescence from allophycocyanin. In *Synechococcus* (Fig. 9c, d), it was less obvious, but present, while the overlap of PBS pigment fluorescence with Chl*a* fluorescence was stronger.

**Table 2:** Fitting criteria for representation of *F*_0_ and *F*_m_ fluorescence using Gaussian curves

Pigment	Gaussian parameter		
	*α*	*β* (nm)	*γ* (nm)
Phycocyanin (PC)	*F*_m_ ≥ *F*_0_ ≥ 0	600–646, *F*_m_ = *F*_0_	10–12, *F*_m_ = *F*_0_
Allophycocyanin (APC)	*F*_m_ ≥ *F*_0_ ≥ 0	655–663, *F*_m_ = *F*_0_	10–12, *F*_m_ = *F*_0_
Chl*a*	*F*_m_ ≥ *F*_0_ ≥ 0	682–685, *F*_m_ = *F*_0_	10–12, *F*_m_ = *F*_0_

When *F*_v_/*F*_m_ data are interpreted in terms of the quantum yield of charge separation in PSII, we assume that observed *F*_v_/*F*_m_ originates fully from Chl*a* located in PSII. This concept is challenged in cyanobacteria where PBS pigment and Chl*a* fluorescence may overlap. Using the Gaussian components of *F*_0_ and *F*_m_, we can express the variable fluorescence of [*F*_v_/*F*_m_]_Chl*a*_ which is the ‘true’ *F*_v_/*F*_m_ that is related to electron transport in PSII. The variable fluorescence that is actually observed is referred to as [*F*_v_/*F*_m_]_obs_. The similarity of [*F*_v_/*F*_m_]_obs_ and [*F*_v_/*F*_m_]_Chl*a*_, where lower values correspond to increased dampening of [*F*_v_/*F*_m_]_obs_ by overlapping pigment fluorescence, can thus be expressed as

2 $$ 1 0 0 {\text{\%}}\,\cdot\,\frac{{[F_{\text{v}} /F_{\text{m}} ]_{\text{obs}} }}{{[F_{\text{v}} /F_{\text{m}} ]_{{{\text{Chl}}a}} }}. $$

**Fig. 10:**
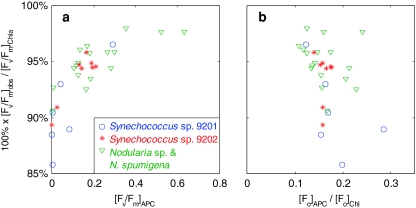
Similarity of [*F*_v_/*F*_m_]_obs_ and [*F*_v_/*F*_m_]_Chl*a*_ (Eq. 2) for cyanobacteria cultures expressed against **a** variable fluorescence originating from allophycocyanin ([*F*_v_/*F*_m_]_APC_) and **b** against the ratio of allophycocyanin-to-Chl*a* *F*_0_. Fluorescence of the individual pigment components was assessed by Gaussian decomposition of *F*_0_ and *F*_m_ emission spectra with excitation at 590 nm

In the absence of phycobilipigments we assume that [*F*_v_/*F*_m_]_Chl*a*_ = [*F*_v_/*F*_m_]_obs_. This was indeed the case for all algal cultures. *B. submarina* gave an average (± standard deviation) similarity of 99.6 ± 0.7% (*n* = 7), and *T. pseudonana* gave 100 ± 1.5% (*n* = 8). The lowest similarity in the set of 31 cyanobacteria cultures was 85.7% for a culture of *Synechococcus* sp. CCY9201 grown for 8 days under medium light in nutrient replete conditions. The average for all cyanobacteria cultures was 93.8 ± 2.9%. This translates to the dampening of a theoretical [*F*_v_/*F*_m_]_Chl*a*_ of 0.65 to [*F*_v_/*F*_m_]_obs_ = 0.61 ± 0.02. We may expect that any combination of low [*F*_v_/*F*_m_]_Chl*a*_, strong PBS fluorescence, and low *F*_v_/*F*_m_ of the PBS pigments leads to a larger deviation of [*F*_v_/*F*_m_]_obs_ from [*F*_v_/*F*_m_]_Chl*a*_. The two latter effects are illustrated in Fig. 10, where the results of Eq. 2 for all cyanobacteria are plotted against the variable fluorescence of the Gaussian component representing allophycocyanin [(*F*_v_/*F*_m_)_APC_, Fig. 10a] and the intensity of *F*_0_ by allophycocyanin relative to Chl*a* [(*F*_0_)_APC_/(*F*_0_)_Chl_, Fig. 10b]. The importance of [*F*_v_/*F*_m_]_APC_ on the similarity between [*F*_v_/*F*_m_]_Chl*a*_ and [*F*_v_/*F*_m_]_obs_ is clear, with the similarity expressed in Eq. 2 decreasing gradually as [*F*_v_/*F*_m_]_APC_ <0.3. The results of Eq. 2 could not be explained by the allophycocyanin-to-Chl*a* *F*_0_ ratio plotted in Fig. 10b. This suggests that the variable fluorescence expressed by the PBS pigments is more important than the cellular pigment ratio in determining [*F*_v_/*F*_m_]_obs_.

### Influence of detector band width and spectral location on retrieval of *F*_v_/*F*_m_

**Fig. 11:**
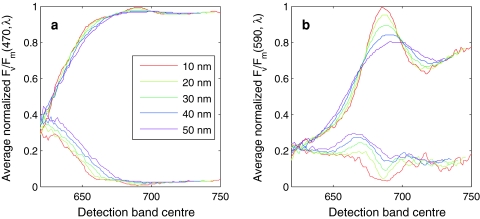
Dampening of observed *F*_v_/*F*_m_ with changing emission band position and width. The plots show the average of *F*_v_/*F*_m_(λ_ex_,λ_em_) measured in all **a** algal cultures, with λ_ex_ = 470 nm, and **b** cyanobacterial cultures, with λ_ex_ = 590 nm. Before averaging, *F*_v_/*F*_m_(λ_ex_,λ_em_) emission spectra were normalized to their peak (found in the 680–690 nm emission region). *Dashed lines* indicate the standard deviation of the normalized *F*_v_/*F*_m_(λ_ex_,λ_em_) emission spectra. All lines were smoothed over 5 nm. The sharply peaked *F*_v_/*F*_m_ feature observed in all cyanobacteria cultures imposes strict limitations on the configuration of the emission slit

The signal-to-noise ratio of a fluorometer improves with increasing width of the emission slit. In addition, shifting the detection band to longer wavelengths reduces cross talk from the excitation source, which becomes important when excitation includes longer wavelengths (e.g. to excite cyanobacterial pigments). The variable fluorescence from cyanobacteria is sharply peaked at the PSII Chl*a* emission band, in contrast to algae (Figs. 5, 7c). The emission detection band width must therefore be sufficiently narrow to retain sensitivity to the optical feature. The effect of the emission bandwidth and spectral location on observed *F*_v_/*F*_m_ is illustrated in Fig. 11. *F*_v_/*F*_m_(590,λ_em_) and *F*_v_/*F*_m_(470,λ_em_) of cyanobacteria and algae cultures, respectively, were normalized to their peak and plotted as a function of λ_em_ between 620 and 750 nm, and for emission band widths ranging 10–50 nm. These spectra are highly conserved between all algae (Fig. 11a), with standard deviation of normalized *F*_v_/*F*_m_ spectra <10% for wavelengths >665 nm (at shorter wavebands coupling of different accessory pigments to PSII introduces some variability). In cyanobacteria (Fig. 11b), the peak of *F*_v_/*F*_m_(590,λ_em_) was sharply defined near the 683-nm peak of PSII Chl*a* emission, with fluorescence from PBS pigments contributing to lower *F*_v_/*F*_m_ on the short wavelength side. At wavelengths >683 nm, non-variable fluorescence from PSI pigments dampens *F*_v_/*F*_m_. Consequently, the observed *F*_v_/*F*_m_ is strongly dependent on the emission detection band centre and width. For broad detection bands positioned >700 nm, the deviation from the maximum *F*_v_/*F*_m_ amounted to up to 35%, equivalent to the reduction of *F*_v_/*F*_m_ = 0.65 as observed for some of our cyanobacteria cultures (Fig. 3) to 0.42. The use of instruments with long-pass filters with a cut-off >700 nm can thus explain low *F*_v_/*F*_m_ readings in cyanobacteria, complementary to the explanation that phycobilipigment fluorescence elevates *F*_0_ as highlighted by Campbell et al. (1998).

### Interpretation of community *F*_v_/*F*_m_ from selected optical configurations

We have demonstrated the need for careful selection of excitation and emission bands in fluorometer design to prevent bias against cyanobacterial representation in the measured signal. We now show some examples of community *F*_v_/*F*_m_ measurements using theoretical fluorometer configurations, using the same simulated community fluorescence data as in preceding exercises. Because we use DCMU instead of illumination-induced *F*_m_ in all simulations, differences in the retrieval of algal or cyanobacterial *F*_v_/*F*_m_ do not reflect the (in)ability of the fluorometer to incite the maximum attainable variable fluorescence. Community *F*_v_/*F*_m_ is, as before, compared to algae- and cyanobacteria-specific *F*_v_/*F*_m_(470,683) and *F*_v_/*F*_m_(590,683), respectively. The excitation bandwidth is indicated for each case, while the emission is recorded in a 10-nm wide band centred at 683 nm, i.e. the optimum setting that allows for cyanobacterial *F*_v_/*F*_m_ values up to the same level as found in algae.

Results for narrow-band (10 nm) single excitation channel configurations with excitation at 470 and 590 nm were already detailed in Fig. 8a, b, respectively. The results for the 470-nm channel configuration (Fig. 8a) were representative of excitation channels throughout the 450–500 nm range (not shown). This configuration is representative of variable fluorescence fluorometers with a filter design similar to those used for the determination of Chl*a* concentration (excitation in the 400–500 nm range, e.g. Corning 5–60 type filter, emission with a high-pass filter >650 nm, e.g. Corning 2–64 filter). Towards longer excitation wavelengths, the representation of cyanobacterial *F*_v_/*F*_m_ increases gradually, first for cultures that produce phycoerythrin and followed by phycocyanin-rich cultures. The 590-nm excitation configuration featured in Fig. 8b is representative of configurations with excitation in the 590–630 nm range, which are not individually shown here. At longer excitation wavelengths >630 nm, fluorescence in both cyanobacteria and algal groups is increasingly excited so that the signal becomes less specific to the cyanobacterial subpopulation. Moving the excitation source from 590 towards 650 nm increases the fluorescence yield in both groups (Fig. 7c), which can be explained by the presence of phycocyanin in all cyanobacterial cultures and the accessory chlorophylls *b* and *c* in the algal cultures. The absorption shoulder of Chl*a* around 625 nm and the main red peak of Chl*a* at 675 nm also increasingly absorb light when the excitation waveband is moved beyond 600 nm (Sathyendranath et al. 1987; Bidigare et al. 1990; Ficek et al*.* 2004). The relatively high affinity for illumination >600 nm in both algae and cyanobacteria implies that the light source need not be as bright to fully saturate PSII in all organisms, and error properties of the *F*_v_/*F*_m_ measurement improve slightly, compared to illumination around 590 nm. At the same time, shorter wavebands prevent crosstalk between excitation and emission bands, an important consideration in fluorometer design.

**Fig. 12:**
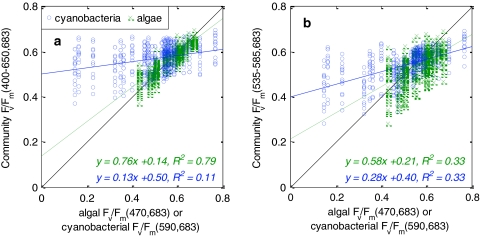
Simulated community *F*_v_/*F*_m_ as a function of algal and cyanobacterial *F*_v_/*F*_m_, for fluorometers with different light source configurations and a 10-nm wide emission band centred at 683 nm. **a** A neutral white light source (400–650 nm), **b** a broad-green light source (535–585 nm)

Results for a fluorometer with broad-white (400–650 nm, spectrally neutral) illumination are given in Fig. 12a. This ‘cool white’ excitation light resulted in a weak representation of cyanobacterial *F*_v_/*F*_m_ against improved results for algal cultures compared to λ_ex_ = 590 nm (Fig. 8b).

Excitation in the 535–585 nm domain should lead to approximately equal representation of algae and cyanobacteria, based on the data shown thus far. Figure 12b shows the result for such a ‘broad-green’ light source. The configuration is still more sensitive to algae than cyanobacteria, but the difference in regression slopes and offsets could at least in part be attributed to the presence of more cases with low *F*_v_/*F*_m_ in the group of cyanobacteria, while scatter is approximately equal for both groups. Cultures of cyanobacteria with low *F*_v_/*F*_m_ (and *F*_0_) had limited influence on community *F*_v_/*F*_m_, especially when paired with healthy algae. For the purpose of identifying community photosynthetic capacity rather than differentiation of algal and cyanobacterial subpopulations, this is not a poor result: phytoplankton that contributes little to community photosynthesis has a proportionally lower impact on community *F*_v_/*F*_m_. This is particularly useful when one is limited to the use of a single-channel fluorometer. This configuration is of special interest because the light source targets exclusively light uptake by accessory photosynthetic pigments in both algae and cyanobacteria (i.e. not Chl*a*), which may render community *F*_v_/*F*_m_ more sensitive to changes in the accessory pigment composition, and thus to environmental conditions.

## Discussion

Cyanobacteria species that are considered harmful due to the production of toxins, odorous compounds, surface scums, or benthic mats, are widespread in coastal and inland water bodies, particularly in eutrophic systems (e.g. Hallegraeff 1993; Anderson et al*.* 2002). Blooms of these species negatively impact ecosystem value. Monitoring the presence and activity of cyanobacteria is therefore a pressing matter in environmental policy. The distinct absorption and fluorescence properties of cyanobacteria caused by the prominent role of phycobilipigments in photosynthetic light harvesting are already used to complement traditional observation methods (e.g. microscope counts) in environmental monitoring (Lee et al. 1994; Izydorczyk et al. 2005; Seppälä et al. 2007). Variable fluorescence measurements are increasingly included in these monitoring efforts, to reveal spatiotemporal trends in photosynthetic capacity or even photosynthetic activity of the phytoplankton.

FRRF instruments equipped with a series of excitation sources are increasingly becoming available, and can be used to determine both the quantum yield of photochemistry and the functional absorption cross-section of PSII at e.g. blue, green and orange or red wavelengths. With these instruments it is possible to better assess the role of phytoplankton that efficiently harvest green and orange light in aquatic photosynthesis in environments where terrigenous organic matter skews the available radiation towards the green part of the light spectrum. Such knowledge may be used to determine ecophysiological constraints of coastal and freshwater phytoplankton, but in a wider sense also help to better represent the role of light uptake in ecosystem models that focus on the environments most exposed to, and most important to, human activities. This progress in FRRF design is made possible through more efficient light sources and detectors that have become available in recent years. It is therefore timely to conceive what properties the optimal instrument for these environments should possess and what pitfalls might be avoided.

Some properties of cyanobacterial fluorescence emission must be taken into account when deciding upon the optimal detection waveband of the fluorometer, and before interpreting fluorescence induction results obtained with different fluorometer configuration. The major light harvesting pigments for photosynthesis in cyanobacteria are organized in the PBS which holds a group of highly fluorescent phycobilipigments. As long as these pigments are organized in the PBS, energy from phycoerythrin and phycocyanin will be transferred towards the core of the PBS where allophycocyanin subsequently fluoresces in the 650–670 nm range. It therefore stands to reason that this spectral domain should be avoided in fluorescence induction measurements where Chl*a* fluorescence is used as a proxy of energy flowing through PSII. Long wavelength (>690 nm) fluorescence from PSI is also relatively strong in cyanobacteria. Regardless of the excitation band that is used we therefore find that narrow (10-nm) wavebands centred at the PSII Chl*a* emission band (680–690 nm) yield best results (Fig. 11).

The efficiency of energy transfer from the PBS to reaction centres is considered very high (Sidler 1994 for a review), but not all harvested energy is transferred to the PSII core. Our results show PBS fluorescence in the order of 22% of *F*_o_ in the Chl*a* emission band. This emission is absent in algae (with exceptions) and theoretically leads to a lowered reading of *F*_v_/*F*_m_ in cyanobacteria and in communities with a high cyanobacterial biomass (Campbell et al. 1996, 1998). We find, however, that a variable component to PBS fluorescence can alleviate the theoretical dampening of *F*_v_/*F*_m_ considerably (Fig. 10). Indeed, the peak of *F*_v_/*F*_m_ in the excitation–emission spectrum is found in the order of 0.65–0.75, for several cyanobacteria species (Fig. 3), despite an average dampening by 6.2% of *F*_v_/*F*_m_ due to the overlapping fluorescence of PBS pigments and Chl*a*. Such high *F*_v_/*F*_m_ values for cyanobacteria have been reported in very few other studies (Raateoja et al. 2004; Suggett et al. 2009), which used FRRF. Variable fluorescence from PBS is surprising; it has been assumed that these pigments do not exhibit variable fluorescence at all. These findings that are reflected in some recent studies using different fluorescence induction techniques (Küpper et al*.* 2009; Kana et al. 2009) challenge the idea of a constant, highly efficient resonance transfer from PBS pigments to the reaction centres. Our fluorescence data provide insufficient means to explore the relation between the rise of PBS fluorescence and closing of PSII reaction centres, or to see how illumination or nutrient conditions might influence PBS *F*_v_/*F*_m_. Nevertheless, it is notable that *F*_v_/*F*_m_ from the PBS at 650 nm showed a fair correlation with cyanobacterial PSII Chl*a* *F*_v_/*F*_m_ (Fig. 8c). In a pilot experiment that is not presented here, we exposed *N. spumigena* with saturating light flashes (590 nm) and observed induction of PBS fluorescence (650 nm), suggesting that the present result is neither merely an artefact of DCMU treatment nor to prolonged exposure to light in our spectrofluorometer. If the mechanism behind phycobilisomal variable fluorescence can be explained in terms of PSII kinetics, this may open up the way to study the physiology of cyanobacteria in natural communities. The possibility of variable fluorescence from phycobilipigments in cryptophytes and rhodophytes should in such studies be taken into account.

The excitation spectrum of fluorescence in PSII is primarily dependent on the photosynthetic pigment composition, which distinguishes the major phytoplankton groups and, with exceptions, clearly separates cyanobacteria from algae (Fig. 2). Blue-green illumination (<550 nm) excites stronger fluorescence in algal cultures than in cyanobacteria (Yentsch and Yentsch 1979; Vincent 1983; Schubert et al. 1989). Longer wavelength illumination favours cyanobacterial fluorescence but algal fluorescence remains significant. If the emission band is located at its optimum of 680–690 nm, as we recommend, the maximum excitation wavelength is practically limited to approximately 650 nm to prevent stray light from the excitation source reaching the detector. There is thus a relatively large section of the photosynthetically active spectrum where algal fluorescence dominates. A ‘white’ illumination source (Fig. 12a), for example, leads to a bias against cyanobacterial representation in community fluorescence. In contrast, a ‘broad-green’ light source (Fig. 12b) that excites predominantly accessory photosynthetic pigments yields near-equal representation of algal and cyanobacterial *F*_v_/*F*_m_. Our results show a relatively low correlation coefficient (*R* ^2^ = 0.33) of the community *F*_v_/*F*_m_ with either group in the community, when we simulate the broad-green light source. Of course, many of the randomly mixed communities combine cultures exposed to widely different growth conditions and with very different *F*_v_/*F*_m_ at a specific excitation-waveband pair, so that the community signal could never represent both subcommunities equally in these cases. The approach of simulating community fluorescence is, therefore, not to be used to interpret fluorometer performance beyond describing how well each group is represented in the community signal. In theory, the broad-green illumination band should predominantly excite accessory photosynthetic pigments, so that those phytoplankton groups that respond positively to the environmental conditions by producing accessory pigments, will dominate the result. This idea warrants further study, particularly in natural environments where such information may be desirable.

For multi-channel configurations, two narrow excitation bands located in the blue and orange-to-red constitute the minimum required combination to resolve some degree of subcommunity variable fluorescence information. Algal variable fluorescence is obtained with high accuracy from the blue channel. The extent to which orange excitation subsequently yields a different *F*_v_/*F*_m_ will give some indication of the variable fluorescence of cyanobacteria in the community. This result is not unambiguous, because equal *F*_v_/*F*_m_ from both blue and orange-excited fluorescence can be interpreted as equal *F*_v_/*F*_m_ in algae and cyanobacteria but also as the absence of fluorescence from cyanobacteria. To differentiate the two cases, the ratio of *F*_0_ intensities of blue versus orange excitation can be used to reveal whether cyanobacteria form a significant part of the community, because the presence of PBS pigment will certainly lead to a markedly higher orange-excited *F*_0_ (results not shown). Of course, this suggested approach is similar to previous attempts to separate phytoplankton groups based on fluorescence excitation spectra (Millie et al*.* 2002; Beutler et al*.* 2002; Beutler et al*.* 2004; Parésys et al*.* 2005; Gaevsky et al*.* 2005; Seppälä and Olli 2008).

The small number of algal and cyanobacterial species used in our experiments, despite being grown in conditions to allow for a wide range in *F*_v_/*F*_m_, limits the applicability of our results. Fluorescence emission profiles of the major algae groups are relatively similar because the main source of fluorescence is always Chl*a* located in PSII. The excitation spectrum, on the other hand, is dependent on the accessory photosynthetic pigments present. The choice of a single chlorophyte and diatom, representing red absorption by Chlorophylls *b* and *c*, is therefore still a realistic representation of many natural communities where algae and cyanobacteria co-exist. It does, however, not cover natural communities extensively. We may consider the case of phycobilin-producing rhodophytes and cryptophytes, as well as cryptophyte-ingesting ciliates (Gustafson et al*.* 2000) in further studies. The fluorescence excitation–emission matrices of rhodophytes are similar to those of the cyanobacteria used here, although planktonic rhodophytes are generally few in environments where cyanobacteria are abundant. We hypothesize that the solutions for instrument design proposed here apply to these algae in the same manner as for the cyanobacteria described here. In contrast, the presence of phycoerythrin in cryptophytes and some dinoflagellates leads to a broader excitation domain in the algal groups. The presence of these ‘special’ algal groups in a natural sample will hamper efforts to decompose multi-channel fluorescence measurements into the contributions by individual groups (but see Seppälä and Olli 2008), even though it should not markedly change our definition of optimal excitation–emission bands to yield results that are most representative of the whole phytoplankton community.

The PBS pigments produced by strains in this study absorb yellow-to-red light as is common to freshwater and coastal species. The presence of oceanic species with forms of phycoerythrin absorbing down to 495 nm (Lantoine and Neveux 1997; Subramaniam et al. 1999; Neveux et al. 2006) would reduce the specificity of the blue-excited fluorescence signals to the algal part of the community, but we remain confident that the inclusion of an orange-to-red excitation band markedly increases sensitivity to the cyanobacteria present. Even with short-wavelength sensitive forms of phycoerythrin present, it is likely that the cyanobacteria will not be equally represented when using a narrow blue light source, which warrants further study.

To our knowledge, the present work constitutes the first effort to relate phytoplankton community variable fluorescence to the contributions from algal and cyanobacterial subpopulations over a wide domain of the spectral excitation–emission matrix. In order to collect this information with a standard, mid-range spectrofluorometer, some allowances have had to be made. We may question whether our analysis, based on dark adapted cells, manipulated in their growth environment to yield a range of *F*_v_/*F*_m_, are representative of results that would be obtained when using actinic light to manipulate *F*_v_′/*F*_m_′. We do believe that transient physiological change (*i.e.* state transitions) observed under (increasing) illumination can contribute to changes in the observed cyanobacterial influence on community variable fluorescence. At the same time, we assume that these changes are not likely to be of such magnitude that they would change our definition of the optimal fluorometer configuration. It would be most useful to see repeat experiments that focus on measuring *F*_v_′/*F*_m_′ under varying actinic light intensities. A quantum-corrected FRRF or PAM instrument operating with multiple excitation bands would be an excellent platform for such investigations, simultaneously eliminating the need to use DCMU to induce *F*_m_.

In conclusion, we observe that microscope-based active fluorescence measurements, flow-cytometry, remote laser stimulated fluorescence and FRRF are examples of emerging methods in oceanography where phytoplankton fluorescence can shed more light on community composition and photosynthetic capacity at the subcommunity level. We foresee that the use of variable fluorescence techniques will gain increasing importance in environmental monitoring as a complementary method to carbon fixation measurements. It is therefore of prime importance to develop instruments and data interpretation techniques that are not biased against any of the major phytoplankton groups, particularly in environments where the physical environment is heterogeneous in time or space, and come to favour one functional group over another. The results presented in this paper will hopefully lead to a standardized and better understood variable fluorescence meter that will support studies of photosynthesis in optically complex environments.
